# The first case of* Escherichia fergusonii* with biofilm in China and literature review

**DOI:** 10.1186/s12879-023-07985-8

**Published:** 2023-01-20

**Authors:** Yi-Ming Zang, Jun-Feng Liu, Gang Li, Mei Zhao, Guo-min Yin, Zheng-ping Zhang, Wei Jia

**Affiliations:** 1 Clinical Medical College, Ningxia Medical University, Yinchuan, China; 2 Medical Experimental Center, General Hospital of Ningxia Medical University, No. 804 Sheng-Li Street, Xing-Qing District, Yinchuan, 750004 Ningxia Hui China; 3 Ningxia Key Laboratory of Pathogenic Microorganisms, Yinchuan, China; 4 Lanzhou University Second Hospital, Lanzhou, China; 5 Department of Radiology, General Hospital of Ningxia Medical University, Yinchuan, China

**Keywords:** Drug resistance, *Escherichia fergusonii*, Infection, Literature review, Silver staining

## Abstract

**Background:**

*Escherichia fergusonii* is a rare opportunistic pathogen in humans and animals, especially with biofilm.

**Methods:**

In one case, *E. fergusonii* with biofilm was detected in the bile, and silver staining was used to prove it had biofilm. The clinical characteristics and drug susceptibility of eight cases of *E. fergusonii* retrieved from the literature were also summarized.

**Results:**

This is a case of *E. fergusonii* with biofilm, which has not been reported in China. The 8 cases retrieved from the literature did not specify whether they had biofilm, but we analyzed their clinical characteristics and drug susceptibility. All patients were treated with antimicrobial drugs. 8 cases showed sensitivity to piperacillin/tazobactam and imipenem in 6 cases (75%), but poor sensitivity to levofloxacin and ciprofloxacin.

**Conclusion:**

The silver staining method proved biofilm in this case, which is the first case of *E. fergusonii* with biofilm in China.

## Introduction

*Escherichia fergusonii* is a gram-negative bacterium belonging to the Genus Escherichia in *Enterobacteriaceae*, which is a rare opportunistic pathogen in humans and animals.

*Escherichia fergusonii* has been isolated from a variety of natural environments, including water [1] and animals [2], as well as from human wound infections, urinary tract infections, bacteremia, diarrhea, pancreatic cancer, and other health conditions [3]. There are even more reports of multiple antibiotic-resistant bacteria [4, 5]. The infection caused by this bacterium cannot be ignored. In this paper, the ERCP (Endoscopic Retrograde Cholangiopancreatography) was performed on a patient with pancreatic head cancer and biliary stones from the General Hospital of Ningxia Medical University in Ningxia Hui Autonomous Region, China. *E. fergusonii* with biofilm was cultured from the bile obtained by ERCP, which has not been reported in China. The clinical characteristics and antibiotic resistance of 8 patients with *E. fergusonii* infections were reviewed and summarized. This was done in order to provide some reference for clinical diagnosis and treatment of this infection.

## Case report

### Case presentation

**Fig. 1:**
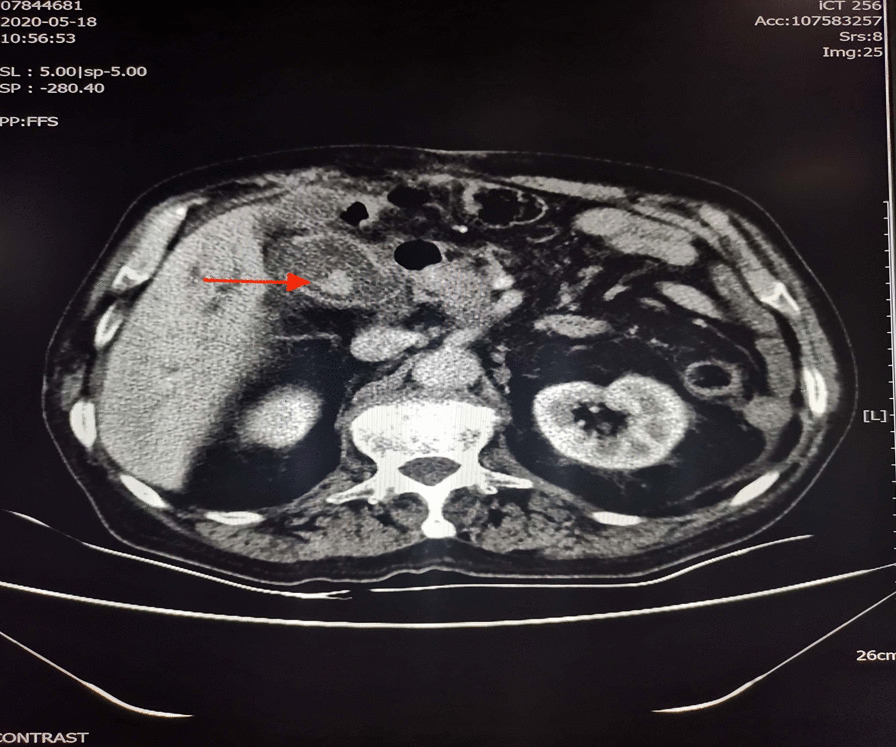
Abdominal CT: enlarged gallbladder, thickened wall and circular high-density shadow

The patient is a 74-year-old male who developed skin and sclera yellow staining with no obvious cause 1 month ago, without fever, chills, abdominal pain, abdominal distension and other discomfort, developed epigastric distension and pain 10 days ago. He had a history of high blood pressure for more than 10 years and denied any history of coronary artery disease or diabetes mellitus. Physical examination shows: the whole abdomen was soft, with Epigastric tenderness, no rebound pain, and muscle tension. The abdominal mass was not touched, Murphy's sign was negative, and the liver and spleen were not touched. His blood count showed a WBC of 8.4 × 10^9^/L, neutrophils of 77.6%, total bilirubin of 77.1 μmol/L, CRP of 97.9 mg/L, PCT of 0.5 ≤ PCT < 2 ng/ml. Abdominal ultrasound suggested: (1) Gallstone; (2) Occupying the lower segment of the common bile duct does not rule out dilation of the hepatobiliary duct; (3) Multiple hilar lymph node enlargements; (4) Peritoneal effusion. Abdominal CT suggested: (1) Gallstones and cholecystitis; (2) Acute pancreatitis; (3) A slight low-density lesion at the head of the pancreas with dilation of the intrahepatic and extrahepatic bile ducts and pancreatic duct was considered to be a malignant tumor (Fig. 1). ERCP + EST (Endoscopic sphincterotomy) + ERPD (Endoscopic pancreatic duct stenting) + EMBE (Endoscopic metal biliary endoprosthesis) + ENBD (Endoscopic nasociliary drainage): the bile duct was naturally shaped, severely dilated, a filling defect was visible, and the lower segment of the common bile duct was interrupted. Maximum diameter of common bile duct 2.0 cm; intrahepatic bile duct was dilated without filling defect. The gallbladder is not visible. After 4 days of empiric treatment with ceftazidime (1.5 g, 12 h/time, iv drip), the patient was discharged after improvement.

### Microbiological examinations

**Fig. 2:**
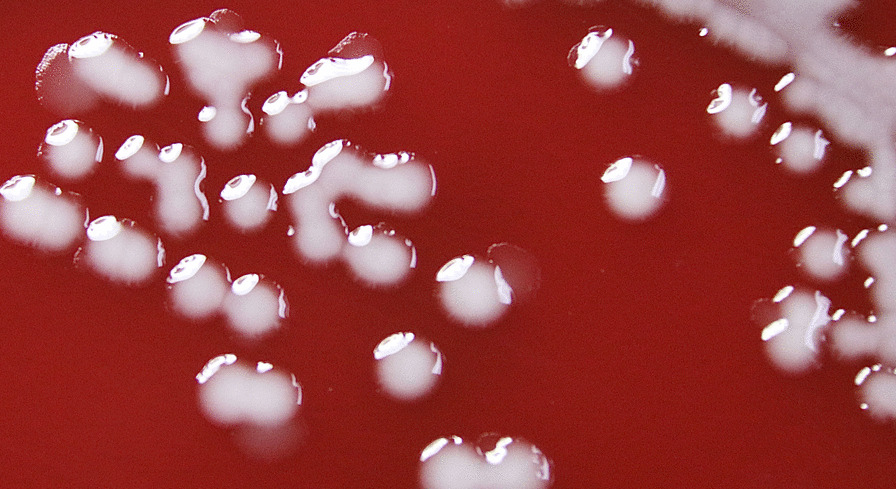
*E. fergusonii* colonies with biofilm on blood agar

The bile was inoculated onto the blood plate and incubated in a 35 ℃ incubator for 24 h. The colony grew on the blood plate as milky white colonies, which grew and fused into gelatinous colonies after 48 h (Fig. 2).

### Bacteria identification

**Fig. 3:**
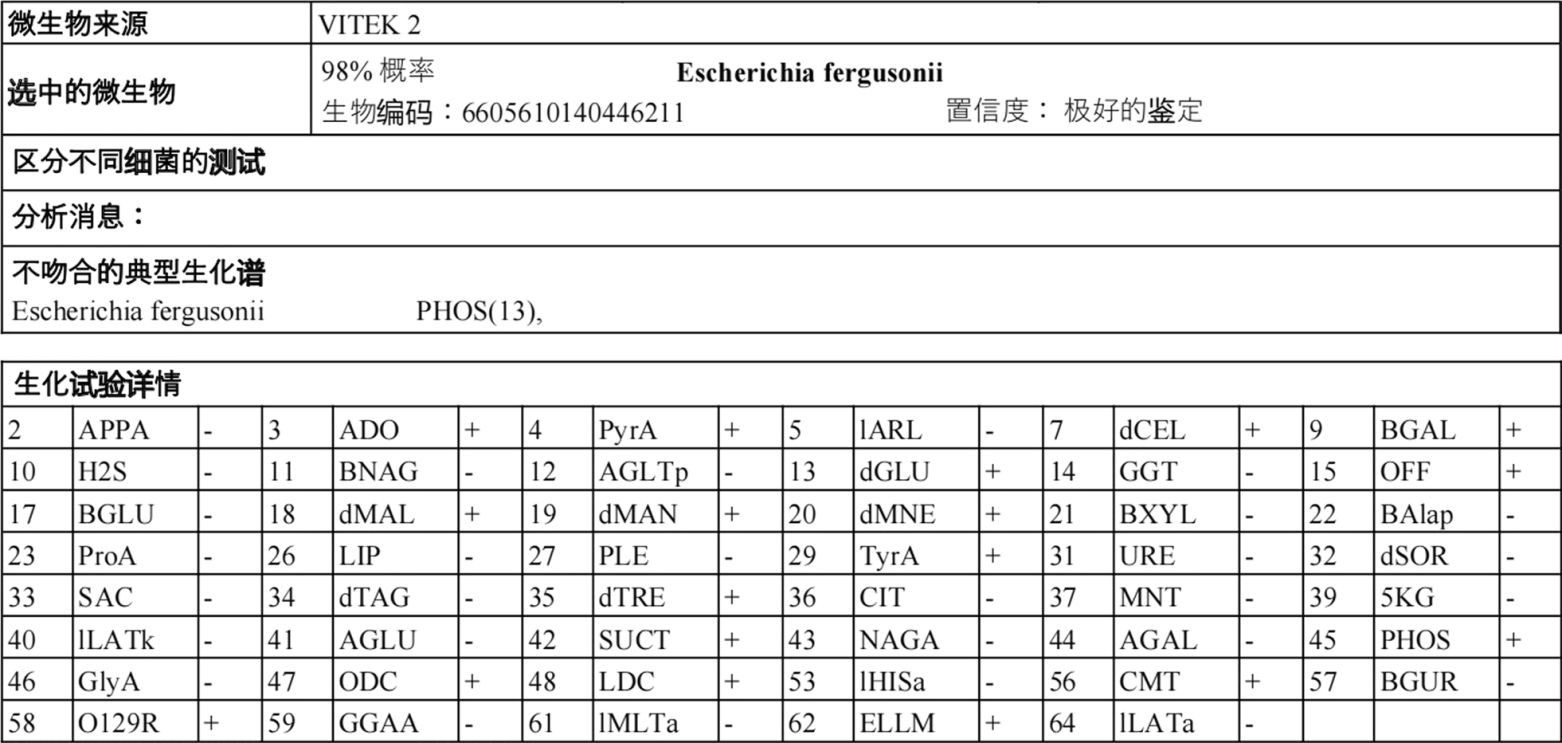
The report of VITEK 2

The identification result of API20E was *E. fergusonii* (99.1%), VITEK 2 was: *E. fergusonii*, biological number: 6605610140446211, confidence: very reliable identification (Fig. 3). The species level was confirmed by 16S rRNA gene sequence analysis. Primers 27F (5′-AGAGTTTGATCCTGGCTCAG-3′) and 1492R (5′-TACGACTTAACCCCAATCGC-3′) were amplified and the resulting product was sequenced. The strain had 99.79% similarity to the 16S rRNA gene of known *E. fergusonii* (NR_027549.1) by logging into the NCBI website and doing similarity analysis with GenBank database by using the BLAST algorithm.

### Silver staining method

**Fig. 4:**
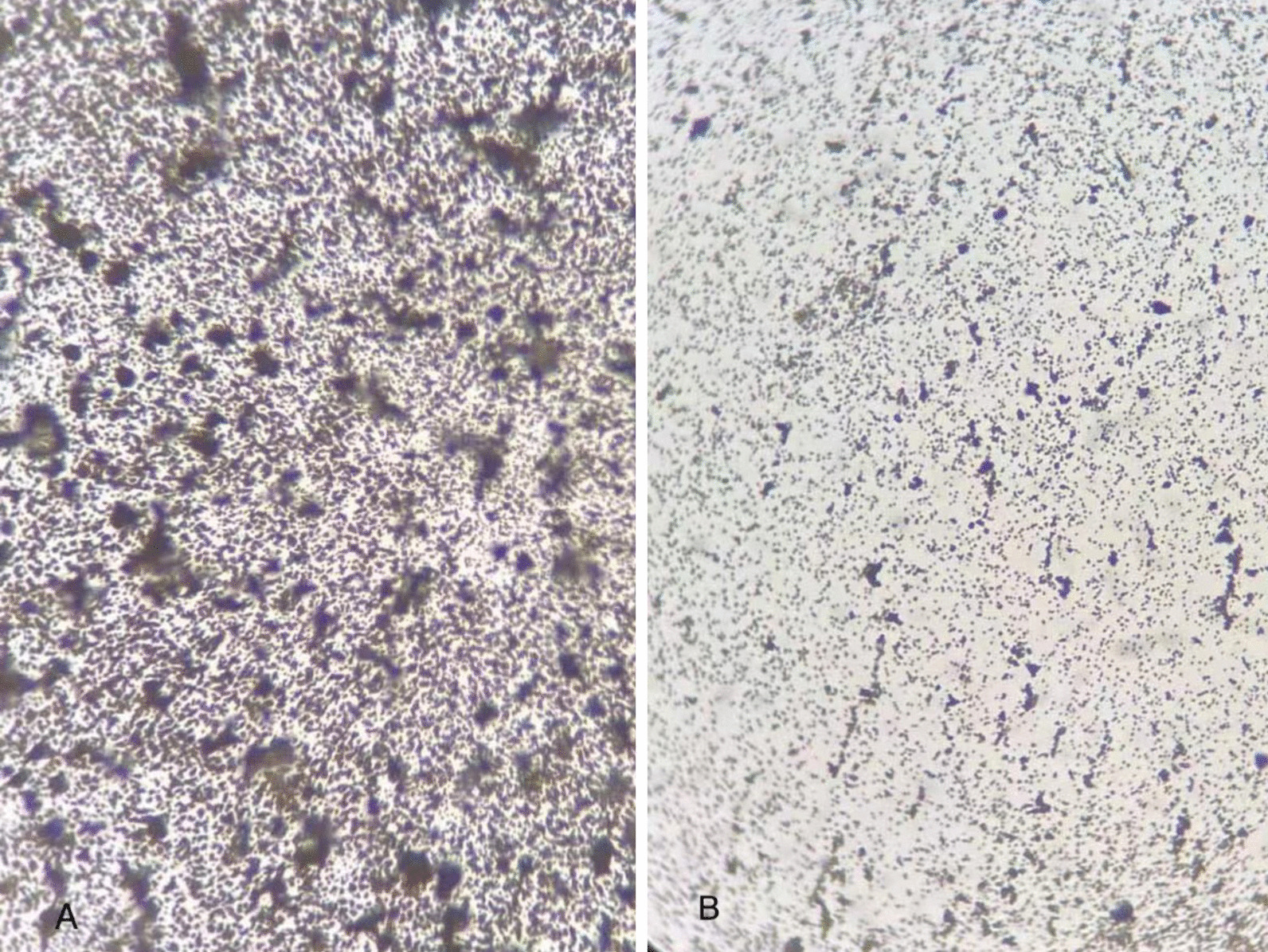
Morphology of this bacteria (**A**) and *E. coli* ATCC8739 (**B**) under microscope, silver staining × 1000

We also applied silver staining to demonstrate the presence of biofilm. A standard strain of *E. coli* (ATCC8739) was used as a negative control. The procedure was performed as follows: (1) put the specimens into 2.5% glutaraldehyde in PBS for 1 h; (2) immersed in distilled water for 1 min; CaCl_2_ soak for 15 min; distilled water for 1 min; 5% AgNO_3_ for 15 min; 1% hydroquinone for 2 min; distilled water for 1 min; 5% NaS_2_O_3_ for 2 min; distilled water for 1 min; (3) air-drying and microscopic observation. Microscopically, bacteria clustered together and polysaccharide protein complexes in the biofilm appeared black brown (Fig. 4).

### Drug susceptibility

Drug susceptibility test was performed by VITEK 2 and the disk diffusion method was used as the supplement test. According to the disk diffusion method recommended by CLSI, put the dried filter paper containing certain antibiotics on the agar plate inoculated with a certain amount of bacteria (0.5 McF), and then observed the size of the bacteriostatic circle after incubation at 35 ℃ for 48 h. Results were determined according to CLSI M100-S30 [6] (Table 1).

**Table 1:** Antibiotic sensitivity results of *Escherichia Fergusonii* search in literature and this case

Case No	TZP	AMS	AMC	AN	ATM	LVX	CIP	CRO	CZ	CAZ	FEP	FM	GM	IPM	SXT	TM
1 [7]	S	–	–	–	–	–	–	–	–	–	–	–	–	S	S	–
2 [8]	S	S	S	S	S	R	R	S	S	S	S	S	S	S	S	S
3 [3]	Unknown															
4 [9]	S	–	S	–	–	–	S	S	-	–	S	–	S	S	–	–
5 [4]	S	–	S	-	R	–	R	R	R	R	S	I	S	–	–	S
6 [5]	S	S	S	–	–	R	R	–	–	S	–	S	R	S	R	R
7 [10]	–	–	–	–	–	–	S	–	–	–	–	S	S	S	S	–
8 [11]	–	–	R	–	–	–	S	S		S	–	–	S	S	–	S
9 (this case)	S	S	–	S	S	I	R	S	S	S	S	S	–	S	S	S

TZP: Piperacillin-tazobactam; AMS: Ampicillin-Sulbactam; AMC: Amoxicillin-clavulanic acid; AN: Amikacin; ATM: Aztreonam; LVX: Levofloxacin; CIP: Ciprofloxacin; CRO: Ceftriaxone; CZ: Cefazolin; CAZ: Ceftazidime; FEP: Cefepime; FM: Nitrofurantoin; GM: Gentamicin; IPM: Imipenem; SXT: Trimethoprim-sulfamethoxazole; TM: Tobramycin

### Treatment

Empirical ceftazidime (1.5 g, twice a day, iv drip) treatment for 4 days.

## Literature review

**Fig. 5:**
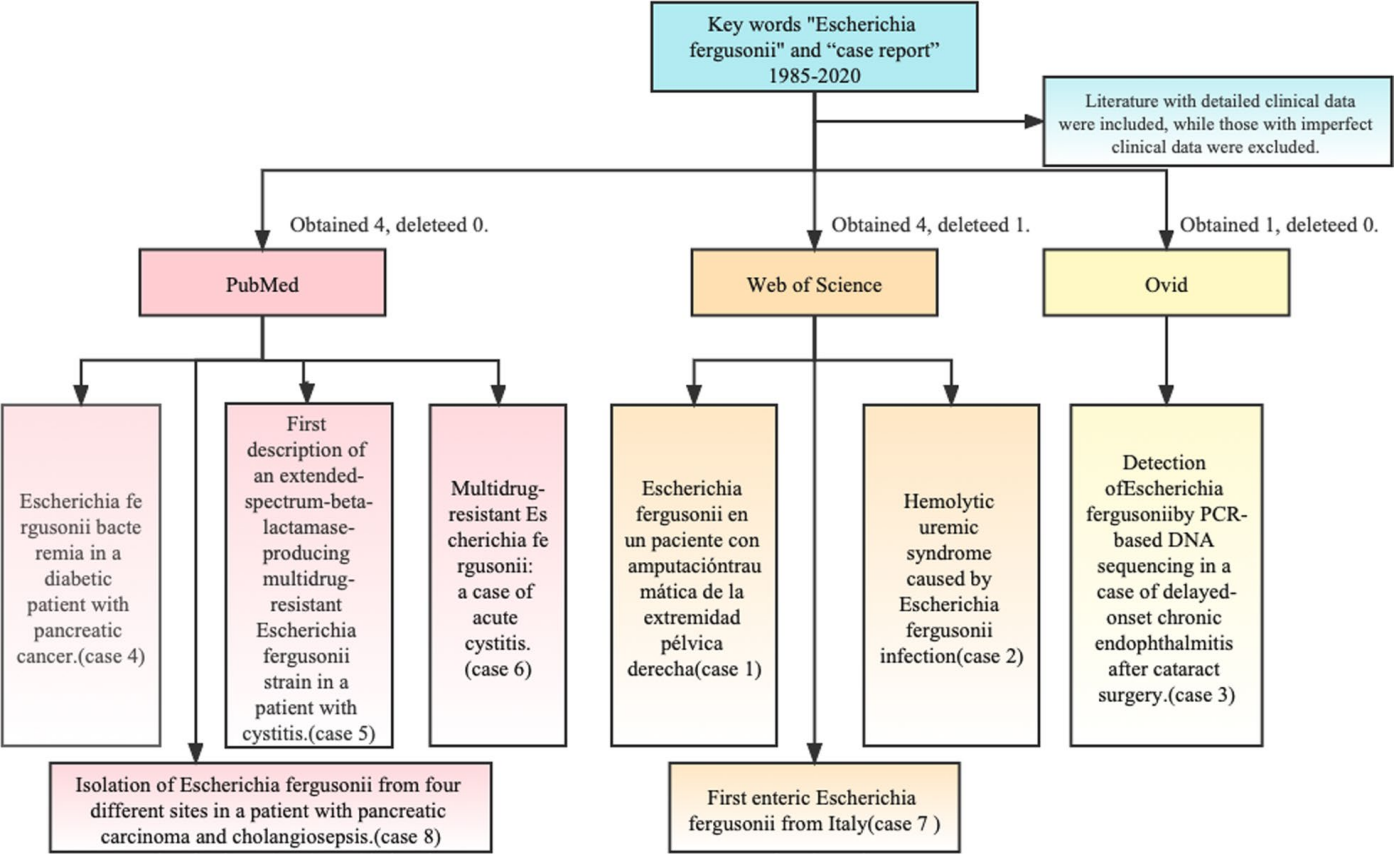
The process of literature search

The PubMed, Ovid, and Web of Science databases were searched by using the advanced search key words "*Escherichia fergusonii*" and “case report” from 1985 to 2020, and those with detailed clinical data were included, while those with imperfect clinical data were excluded (Fig. 5). A total of 8 cases (4 cases from PubMed, 3 cases from Web of Science, 1case from Ovid) were included in summary (Table 2). In these eight cases, it was not specified whether the detected *E. fergusonii* had a biofilm and whether biofilm testing was performed. However, we analyzed their clinical characteristics and drug susceptibility to provide some support for future treatment.

**Table 2:** Relevant case data of *Escherichia Fergusonii* in literature and this case

Case No	Age/sex	Region	Method	Underlying diseases	Source of specimen	Infection	Therapeutic regimen	Surgical treatment	Outcome
1 [7]	12/F		API VITEK MS VITEK 2	No	The tissue and bone of the stump	Osteomyelitis	ETP and AM, 1 month	Yes	Recovered
2 [8]	76/F	Korea	Unknown	Diabetes, stroke	Urine, blood	Urinary tract infection, acute kidney injury, hemolytic uremic syndrome	CRO, 26 days; CRRT	No	Recovered
3 [3]	72/F	India	PCR	cataract surgery, systemic steroids	Anterior chamber puncture fluid	Hypopyon uveitis	VA, CAZ (intravitreal injections); vitrectomy with IOL removal	Yes	Improved
4 [9]	73/M	Taiwan, China	VITEK 2 16s RNA	Diabetes mellitus, pancreatic cancer	Blood	Fischemic bowel diseases, severe sepsis	CRO, 7 days	No	Died
5 [4]	76/F	Canada	VITEK 2	hip arthroplasty, Foley Catheter, traveler’s diarrhea	Urine, stool	Catheter-associated cystitis, Intestinal infection	SXT, 3 days; AMC, 7 days	No	Died
6 [5]	52/F	Italy	VITEK 2	No	Urine	acute cystitis	Sequential replacement: CFM, 1 week; CTX and FM, 2 weeks	No	Cured
7 [10]	M	Italy	API VITEK 2 16s RNA	Leukaemic	Stool	Without signs of enteric infection	Unknown	No	Unknown
8 [11]	69/M	Switzerland	API	Pancreatic Carcinoma, Cholangiosepsis	Bile, blood, stool, Abdominal wound secretion	Bloodstream infection, Biliary infection	AMC, TM, IPM	No	Died
9 (this case)	74/M	Ningxia, China	VITEK 2 16s RNA	Pancreatic head cancer, Biliary stones	Bile	Biliary infection	CAZ, 4 days	Yes	Recovered

Cured: symptoms disappeared and the inflammatory indicators returned to normal with medication. Recovered: symptoms disappeared with medication but laboratory indicators did not return to normal or were not mentioned. Improved: symptoms disappear with non-pharmacological treatment such as surgery

ETP: Ertapenem; AM: Ampicillin; CRO: Ceftriaxone; VA: Vancomycin; CAZ: Ceftazidime; SXT: Trimethoprim-sulfamethoxazole; AMC: Amoxicillin-clavulanic; CFM: Cefixime; CTX: cefotaxime; FM: nitrofurantoin; TM: tobramycin; IPM: Imipenem; CRRT: Continuous Renal Replacement Therapy; EST: Endoscopic Sphincterotomy; ERPD: Endoscopic Pancreatic Duct Stenting; EMBE: Endoscopic Metal Biliary Endoprosthesis; NBD: Endoscopic nasociliary drainage

## Discussion

*Escherichia fergusonii* is a rare opportunistic pathogen in humans and animals. In 1985, Farmer et al. [12] separated and confirmed that *E. fergusonii* was a newly discovered species of *Enterobacteriaceae* in the blood samples of clinical patients. Before that, it was known as group 10 of intestinal bacteria in general. In 1991, Lawrence et al. [13] found that the bacterium had the closest genetic relationship with *E. coli*, which also belonged to *E. coli*, and DNA hybridization showed that they had 64% similarity. Compared with *E. coli*, there are few reports of patients infected with *E. fergusonii*. Due to the rarity of *E. fergusonii*, many conventional laboratories cannot distinguish *E. coli* and *E. fergusonii* [14].

In 1993, Funke et al. [11] separated *E. fergusonii* from bile, blood, feces, and abdominal wound secretion of a 69-year-old male patient with pancreatic cancer and bile duct abscess. However, it was confirmed to be the same bacterial clone after experimental analysis. It has been reported that this bacterium was detected in feces before [4, 10], and it is well known that the intestinal tract is connected with the biliary duct and pancreatic duct, so it is not clear whether *E. fergusonii* migrates from the intestinal tract to the bile duct to cause infection or the bile specimens collected are contaminated with intestinal contents. In 2011, Lai et al. [9] from Taiwan in China detected this bacterium in the blood of a 73-year-old male patient with pancreatic cancer, but not in feces and bile. The patient, in this case, was a 74-year-old male patient with pancreatic head cancer and biliary stones, and bile samples were aspirated by an endoscopically aseptic catheter during ERCP surgery, which could basically rule out the possibility of intestinal contamination. Unfortunately, fecal bacteria were not collected for culture and cannot be traced further. Whether this bacterium has a certain organ-targeting for the cancerous tissues of pancreatic cancer remains to be further studied, but when biliary tract infection occurs in elderly patients with pancreatic cancer, this bacterium should be excluded.

Farmer et al. [12] isolated 2 strains of *E. fergusonii* from human blood for the first time, and collected a total of 25 strains from the American Strain Preservation and Management Center, including 2 strains from blood, 5 strains from urine, 1 strain from abdominal infection, 16 strains from feces and 1 strain from others, but lacked more detailed clinical data. This group reviewed 8 patients, with all eight cases being *E. fergusonii*: male: female = 5:3, average age 61.4 years old, ≥ 60 years old accounted for 62.5%, with 75% having basic diseases, including malignant tumor disease (2 cases, 25%), diabetes mellitus (2 cases), surgery or trauma (2 cases), biliary tract disease (1 case, 12.5%), stroke (1 case). The infection sites were urinary tract infection, blood flow infection, and intestinal infection in 3 cases, and bone tissue, secretion, and abdominal wound infection in 1 case. The source of the specimen was almost identical to the site of infection. 8 strains were identified by API, VITEK2, and VITEK MS, all of which can be used to detect *E. fergusonii*. In this case, the strain was identified by VITEK 2 and then verified by 16s RNA.

All of these 8 patients were treated with antibiotics through empirical or drug susceptibility results. Cephalosporins and quinolones were most commonly used, and most of them were improved or cured (4 cases, 50%). 3 cases died due to serious underlying diseases combined with infection, and 1 case was unknown. Drug susceptibility revealed that *E. fergusonii* was most sensitive to Piperacillin/tazobactam and Imipenem, but less sensitive to Levofloxacin and Ciprofloxacin. In 2005, India Mahapatra et al. [15] isolated 104 strains of *E. fergusonii* from 600 clinical specimens, 83% of which were susceptible to amikacin, followed by Cefoperazone/sulbactam (79%) and Gatifloxacin (73%), Cefotaxime and Ciprofloxacin 53% and 33%, respectively. Gentamicin showed the poorest sensitivity. However, the susceptibility to Imipenem was not mentioned in the report. With the emergence of multi-antibiotic resistant *E. fergusonii*, it is vital that we evaluate the antibiotic sensitivity of more cases in future research.

The biofilm is a structured community of bacteria that adheres to a contact surface. It secretes polysaccharide matrix and other extracellular polymers, and encapsulates itself in a large number of bacterial aggregates [16]. Studies have shown that bacteria in biofilms are 10–1000 times more resistant to antibiotics than bacteria in their planktonic state, which can easily lead to the development of bacterial drug resistance and cause serious clinical problems, resulting in recurrent attacks of many chronic and refractory infections. The increasing number of drug-resistant strains of *E. coli*, which also belong to the *Enterobacteriaceae*, is mostly due to the formation of its biofilm. *E. coli* with biofilm helps it to resist conditions such as oxidative stress, antibiotic treatment, dehydration and starvation [17]. Scientists believe that biofilm resistance may be related to nutritional restriction, impaired antibiotic penetration, inhomogeneity of the biofilm, abnormal expression of efflux pump genes, horizontal transfer of genes, and secretion of inactivating enzymes.

However, due to the rarity of *E. fergusonii* with biofilm, its resistance mechanism and other related mechanisms need to be further studied.

## Conclusion

While the 8 cases retrieved from the literature did not specify whether they had a biofilm, the discussion suggests it may have colonized clinical patients or the environment, and immunocompromised patients and seniors with certain underlying diseases are at increased risk of infection. Moreover, as this is the first case of *E. fergusonii* with biofilm in China, its pathogenetic mechanisms and other related studies need to be investigated further.

## Data Availability

All data generated or analyzed during this study are included in this published article (and its supplementary information files).
